# Comparison of early clinical and optical quality-related outcomes after SMILE using VisuMax 800 versus VisuMax 500 for myopia and low-to-moderate astigmatism in a Chinese population: a single-center retrospective study

**DOI:** 10.3389/fmed.2026.1790038

**Published:** 2026-04-30

**Authors:** Xuanyu Yang, Jiabin Leng, Yuchen Mei, Fei Peng, Yifan Xie, Linlin Liu, Linhua Wan, Liping Liu, Wentian Zhou, Hui Zhang

**Affiliations:** 1 Nanchang Bright Eye Hospital, Nanchang, Jiangxi, China; 2 School of Ophthalmology and Optometry, Jiangxi Medical College, Nanchang University, Nanchang, Jiangxi, China; 3 School of Statistics and Data Science, Jiangxi University of Finance and Economics, Nanchang, China

**Keywords:** astigmatism, centration, higher-order aberrations, small incision lenticule extraction, SMILE, SMILE Pro, VisuMax 800

## Abstract

**Purpose:**

To compare early clinical outcomes, centration accuracy, and optical quality–related parameters after small incision lenticule extraction (SMILE) performed using the VisuMax 800 and VisuMax 500 platforms in a Chinese population with myopia and low-to-moderate astigmatism.

**Methods:**

This retrospective, single-center comparative study included 200 patients (200 eyes) who underwent SMILE between May and August 2025. Patients were grouped according to the laser platform used in routine clinical practice: the SMILE group (VisuMax 500, *n* = 100) and the SMILE Pro group (VisuMax 800, *n* = 100). To avoid inter-eye correlation, one eye per patient was included in the analysis. Baseline characteristics were comparable between groups. At 3 months postoperatively, outcomes assessed included visual acuity, refractive outcomes, optical zone decentration, corneal higher-order aberrations, astigmatism vector parameters using the Alpins method, and exploratory correlation analyses between centration metrics and postoperative optical and astigmatic outcomes.

**Results:**

No significant between-group differences were observed in postoperative uncorrected or corrected distance visual acuity, safety index, or efficacy index (all *p* > 0.05). Compared with the SMILE group, the SMILE Pro group showed significantly shorter lenticule creation time (9.47 ± 0.88 s vs. 27.43 ± 2.26 s, *p* < 0.01), lower absolute Y-axis decentration and total optical zone decentration (both *p* < 0.01), and lower postoperative vertical coma (*p* = 0.004). Postoperative residual cylindrical error was also lower in the SMILE Pro group (*p* = 0.048), although the absolute between-group difference was small. In astigmatism vector analysis, the SMILE Pro group showed lower magnitude of error and index of success values (both *p* < 0.05). Exploratory correlation analysis showed a significant positive association between vertical decentration and postoperative vertical coma in both groups, whereas total decentration was significantly associated with the index of success only in the SMILE group.

**Conclusion:**

At 3 months postoperatively, both VisuMax 500-and VisuMax 800-based SMILE showed favorable safety and efficacy for the correction of myopia and low-to-moderate astigmatism. Compared with conventional SMILE (VisuMax 500), SMILE Pro (VisuMax 800) was associated with shorter laser scanning time, lower optical zone decentration, lower postoperative vertical coma, and more favorable astigmatic vector outcomes. These findings represent early postoperative results and should be interpreted cautiously. Further prospective, multicenter studies with larger sample sizes and longer follow-up are warranted.

## Introduction

Keratorefractive lenticule extraction (KLEx) refers to a group of corneal refractive procedures in which an intrastromal lenticule is created and extracted to correct refractive error (1). Small incision lenticule extraction (SMILE) is a flapless corneal refractive procedure in which a femtosecond laser creates a stromal lenticule with anterior and posterior surfaces within the cornea. The lenticule is then dissected and extracted through a small peripheral corneal incision to correct refractive error and improve uncorrected visual acuity (2–4). Because SMILE does not require flap creation, it may help preserve corneal biomechanics and reduce some flap-related complications while maintaining favorable efficacy, safety, and predictability (5, 6). Owing to these characteristics, SMILE has become an established refractive surgical technique in the management of myopia and myopic astigmatism.

Although SMILE has been shown to be effective, safe, and stable over the long term (7), several practical intraoperative and postoperative issues may still occur when the procedure is performed with the conventional VisuMax 500 platform. These include suction loss, cap-related complications, lenticule decentration, refractive undercorrection or regression, and other postoperative complications (8). To address some of these limitations, the second-generation femtosecond laser platform VisuMax 800 was introduced. Compared with the VisuMax 500, the VisuMax 800 provides a substantially higher repetition rate and shorter laser scanning time, and incorporates intelligent assistance systems for centration and cyclotorsion compensation. Early clinical reports have shown that SMILE Pro performed with the VisuMax 800 is safe and effective for the correction of myopia and myopic astigmatism (9). A comparative study by Kim and Chung (10) reported no significant between-group differences in early visual acuity or refractive outcomes between the VisuMax 500 and VisuMax 800 platforms, whereas decentration and the induction of some higher-order aberrations were lower with the VisuMax 800 platform.

However, evidence regarding centration-related outcomes, corneal aberration-related parameters, and astigmatism vector outcomes in Chinese patients remains limited, particularly in direct comparison with the conventional VisuMax 500 platform. To the best of our knowledge, this study is among the early Chinese cohorts to directly compare clinical outcomes and optical quality–related parameters between SMILE performed with the VisuMax 800 and VisuMax 500 platforms. We aimed to compare the two platforms with respect to optical zone decentration, refractive outcomes, safety, efficacy, corneal higher-order aberrations, and astigmatism vector parameters. In addition, we performed exploratory correlation analyses to assess the relationship between centration-related parameters and postoperative optical and astigmatic outcomes. We hypothesized that the VisuMax 800 platform might be associated with improved centration-related outcomes and lower induction of some aberration-related parameters, but these associations were interpreted cautiously.

## Methods

### Study design and participants

This retrospective, single-center comparative study reviewed the medical records of patients who underwent small incision lenticule extraction (SMILE) for myopia or compound myopic astigmatism at Nanchang Bright Eye Hospital during the study period of May to August 2025. A total of 200 patients were included, with 100 patients in the conventional SMILE group and 100 patients in the SMILE Pro group. Patients were grouped according to the laser platform used in routine clinical practice: the conventional SMILE group was treated with the VisuMax 500 system (Carl Zeiss Meditec, Jena, Germany), and the SMILE Pro group was treated with the VisuMax 800 system (Carl Zeiss Meditec, Jena, Germany). Both patient enrollment and surgery were completed during the same study period, and the two laser platforms were used concurrently in routine clinical practice. Group allocation was not randomized. All procedures were performed by the same experienced surgeon (Hui Zhang), which minimized inter-surgeon variability. However, because of the retrospective design, potential selection bias and residual learning-curve effects could not be completely excluded.

The inclusion criteria were as follows: age 18 to 30 years; preoperative manifest spherical error of −6.00 D or less; preoperative manifest cylindrical error of −2.00 D or less; central corneal thickness of at least 500 μm; predicted residual stromal bed thickness of at least 300 μm; and preoperative corrected distance visual acuity (CDVA) of 20/30 or better. Exclusion criteria included severe dry eye disease, keratoconus or suspected corneal ectasia, progressive refractive instability, active ocular disease, lens opacity, ocular infection, or other ocular conditions that could affect visual or refractive outcomes.

Only patients who completed the entire 3-month postoperative follow-up were included in the final analysis. Because both eyes underwent surgery in many patients, only one eye per patient was included to avoid inter-eye correlation. The study eye was selected using a random number table: odd numbers corresponded to the left eye and even numbers to the right eye.

This study adhered to the tenets of the Declaration of Helsinki and was approved by the Institutional Review Board of Nanchang Bright Eye Hospital (Protocol No. 202512–02). Written informed consent was obtained from all participants according to institutional review board policy.

### Surgical procedures

All procedures were performed by the same experienced surgeon (Hui Zhang). The intended postoperative refraction was plano in all eyes. Topical 0.5% lidocaine hydrochloride was used for anesthesia in all cases.

In the conventional SMILE group, surgery was performed using the VisuMax 500 platform. Patients were instructed to fixate on the coaxially positioned target light, and centration was achieved intraoperatively during docking according to the surgeon’s standard alignment procedure. After suction was applied, the femtosecond laser created the posterior lenticule surface, lenticule side cut, anterior cap interface, and cap side cut, followed by standard lenticule dissection and extraction.

In the SMILE Pro group, surgery was performed using the VisuMax 800 platform operating at 2,000 kHz, with a pulse energy of 135 nJ. During docking, the CentraLign guidance system was used for centration. In addition, OcuLign cyclotorsion compensation was enabled in all SMILE Pro eyes.

Refractive treatment planning in both groups was based on the manufacturer-recommended nomogram. No additional surgeon-specific manual adjustment or customized platform-specific nomogram modification was applied during the study period. Thus, each eye was treated according to the standard nomogram setting recommended for its respective laser platform. Geometric treatment settings were kept comparable between platforms whenever possible.

The lenticule dissection and extraction procedure, as well as the postoperative medication regimen, were otherwise identical between the two groups. Because cyclotorsion compensation may influence astigmatic outcomes, only eyes with preoperative refractive cylinder of 2.00 D or less were included in the present study to reduce the confounding effect of larger astigmatic corrections.

### Outcome measures

Postoperative examinations were performed at 3 months. The primary outcome was total optical zone decentration at 3 months postoperatively. Secondary outcomes included uncorrected distance visual acuity (UDVA), corrected distance visual acuity (CDVA), postoperative spherical equivalent, postoperative refractive cylinder, efficacy index, safety index, corneal higher-order aberrations, and astigmatism vector parameters. Exploratory analyses included correlations between decentration metrics and postoperative vertical coma, as well as between decentration metrics and the index of success.

Optical zone decentration was measured using Pentacam (Oculus, Wetzlar, Germany) under dark-room conditions. The reference point for decentration analysis was set as the corneal vertex. Horizontal decentration, vertical decentration, and total decentration were recorded for each eye.

Corneal higher-order aberrations, including total higher-order aberrations, spherical aberration, horizontal coma, and vertical coma, were assessed over a 6-mm optical zone using Pentacam. Standard refractive surgery reporting graphs were generated to evaluate efficacy, safety, predictability, and stability. Astigmatism vector analysis was performed according to the Alpins method, and the corresponding polar plots were generated using Visualize software (version 1.1).

### Statistical analysis

Statistical analyses were performed using Python software (version 3.11.9; Python Software Foundation, Beaverton, OR, United States). Continuous variables are presented as mean ± standard deviation (SD), and categorical variables are presented as counts and percentages. Visual acuity was analyzed in decimal notation. For the principal between-group comparisons, mean differences and 95% confidence intervals were additionally reported.

Normality was assessed using the Shapiro–Wilk test, and homogeneity of variance was assessed using Levene’s test. Between-group comparisons were performed using the independent-samples *t* test for continuous variables and the chi-square test for categorical variables. Within-group preoperative-postoperative comparisons were performed using the paired-samples *t* test. Correlation analyses between decentration metrics and postoperative vertical coma or index of success were performed using Pearson correlation analysis. All statistical tests were two-sided, and *p* < 0.05 was considered statistically significant.

Because of the retrospective design, no *a priori* sample size calculation or formal power analysis was performed. The correlation analyses were exploratory in nature and should be interpreted accordingly.

## Results

### Baseline characteristics

A total of 200 eyes from 200 patients were included in the final analysis, with 100 eyes in the SMILE group (VisuMax 500) and 100 eyes in the SMILE Pro group (VisuMax 800). All patients completed the 3-month postoperative follow-up. As summarized in Table 1, there were no statistically significant differences between the two groups with respect to baseline demographic or preoperative ocular parameters, including age, sex distribution, intraocular pressure (IOP), spherical error, cylindrical error, the distribution of astigmatism, spherical equivalent (SE), keratometric values (K1 and K2), central corneal thickness (CCT), optical zone diameter, uncorrected distance visual acuity (UDVA), corrected distance visual acuity (CDVA), pupil diameter, pupillary offset, or preoperative higher-order aberrations (all *p* > 0.05).

**Table 1:** Comparison of baseline characteristics and preoperative ocular parameters between the SMILE and SMILE Pro groups.

Characteristic	SMILE	SMILE Pro	*p*-value
Patients, eyes	100/100	100/100	N/A
Age	22.36 ± 3.50	22.41 ± 3.03	0.931
Gender	44/56	44/56	1.000
IOP	17.29 ± 2.49	17.04 ± 1.91	0.427
Refractive errors			
Spherical	−3.34 ± 1.38	−2.98 ± 1.22	0.054
Cylindrical	−0.945 ± 0.51	−0.945 ± 0.57	1.000
AX	178.32 ± 29.21	4.2 ± 26.68	0.193
SE	−3.81 ± 1.39	−3.46 ± 1.24	0.057
UDVA	0.14 ± 0.12	0.15 ± 0.12	0.504
CDVA	1.19 ± 0.11	1.20 ± 0.10	0.490
Corneal morphology			
K1	42.30 ± 1.06	42.00 ± 1.41	0.077
K2	43.08 ± 1.44	43.13 ± 7.4	0.943
CCT	555.47 ± 23.68	559.03 ± 27.00	0.323
Optic zone	6.77 ± 0.15	6.77 ± 0.18	0.795
Preoperative pupillary offset			
X-axis	0.15 ± 0.09	0.14 ± 0.10	0.434
Y-axis	0.16 ± 0.12	0.17 ± 0.11	0.540
Pupil diameter	6.85 ± 0.58	6.76 ± 0.59	0.253
HOAs			
Spherical aberration	0.27 ± 0.02	0.27 ± 0.12	0.744
Horizontal coma	0.14 ± 0.11	0.13 ± 0.09	0.483
Vertical coma	0.17 ± 0.10	0.16 ± 0.09	0.458
Total HOAs	0.40 ± 0.12	0.40 ± 0.09	0.986
Horizontal trefoil (0°)	0.02 ± 0.02	0.025 ± 0.03	0.168
Oblique trefoil (30°)	0.03 ± 0.02	0.04 ± 0.11	0.273

Data are presented as mean ± standard deviation (SD), except for gender which is presented as counts. Descriptive statistics for the astigmatism axis were calculated using circular statistics (circular mean ± circular SD). IOP, intraocular pressure; D, diopters; SE, spherical equivalent; UDVA, uncorrected distance visual acuity (decimal); CDVA, corrected distance visual acuity (decimal); K, keratometry; CCT, central corneal thickness; HOAs, higher-order aberrations. Statistical Analysis: Differences between the SMILE and SMILE Pro groups were evaluated using independent two-sample *t*-tests for continuous variables and chi-square tests for categorical variables. Comparisons for astigmatism axis were performed using circular statistical methods. No statistically significant differences were observed at baseline (*p* > 0.05).

### Intraoperative findings

Intraoperative parameters are presented in Table 2. No intraoperative complications, including suction loss or cap-related events, were observed in either group. The time required for lenticule creation was significantly shorter in the SMILE Pro group (VisuMax 800) than in the SMILE group (9.47 ± 0.88 s vs. 27.43 ± 2.26 s, *p* < 0.01). No statistically significant difference was found between groups in the time required for lenticule dissection and removal (*p* > 0.05).

**Table 2:** Comparison of intraoperative parameters and centration metrics between the SMILE and SMILE Pro groups.

Parameter	SMILE	SMILE Pro	*p*-value
Intraoperative complication	0	0	N/A
Time for lenticule creation	27.43 ± 2.26	9.47 ± 0.88	< 0.01
Time for lenticule removal	33.62 ± 10.82	31.94 ± 7.75	0.209
Optic zone decentration			
Absolute X-axis decentration	0.2 ± 0.15	0.16 ± 0.12	0.053
Absolute Y-axis decentration	0.31 ± 0.22	0.13 ± 0.10	<0.01
Total decentration	0.41 ± 0.21	0.23 ± 0.12	<0.01

Data are presented as mean ± standard deviation (SD). N/A, not applicable (due to zero variance/events); s, seconds; mm, millimeters. Statistical Analysis: Differences between groups were evaluated using independent two-sample *t*-tests. Statistically significant differences (*p* < 0.05) are observed in lenticule creation time, absolute Y-axis decentration, and total decentration.

### Primary outcome: optical zone decentration

**Figure 1:**
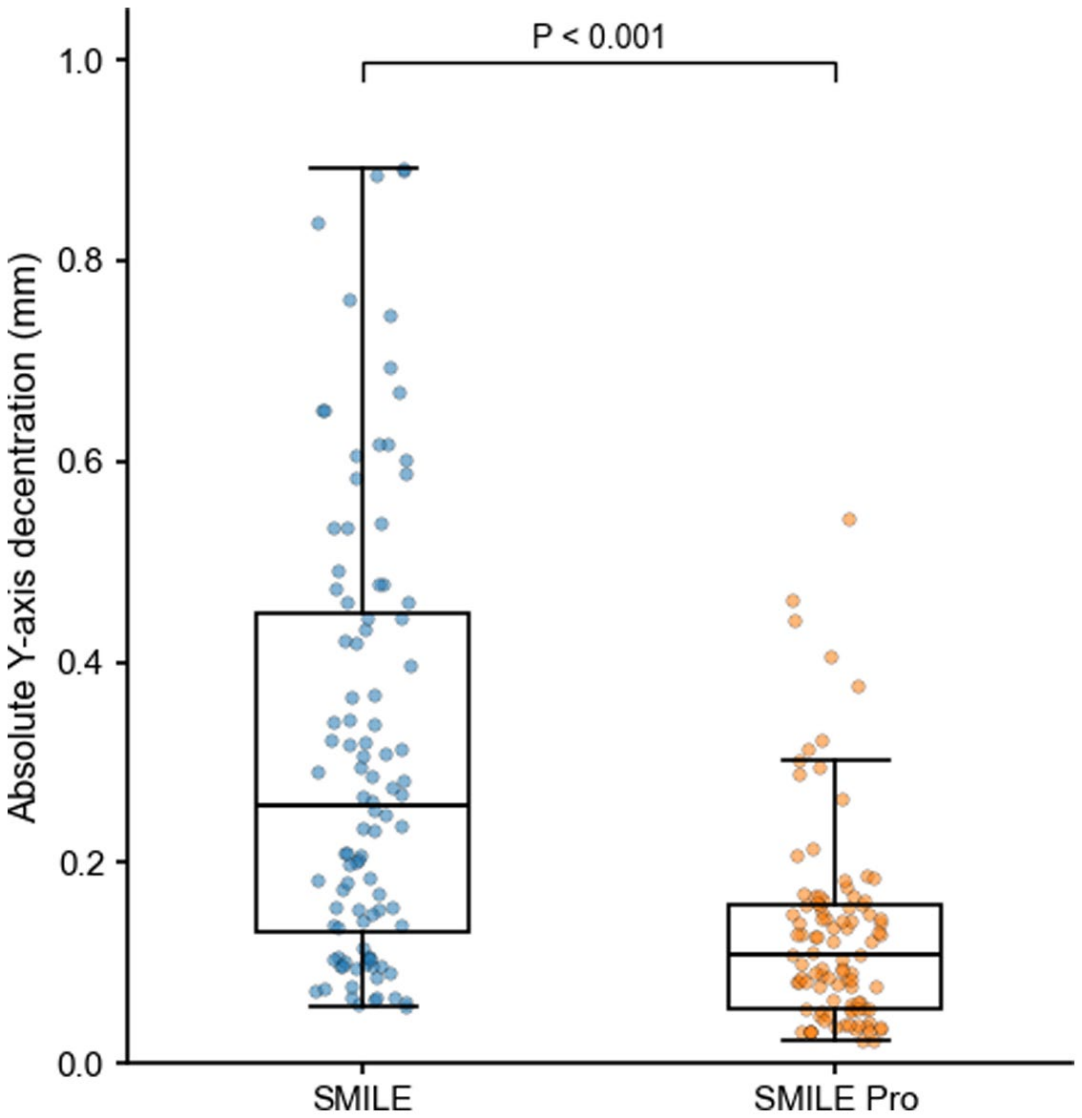
Absolute Y-axis optical zone decentration in the SMILE and SMILE Pro groups at 3 months postoperatively. Data are shown as box plots with individual data points. Absolute Y-axis decentration was measured relative to the corneal vertex.

With respect to centration metrics, optical zone decentration vectors in both groups showed a predominant nasal distribution. The primary outcome, total optical zone decentration, was significantly lower in the SMILE Pro group (VisuMax 800) than in the SMILE group (0.23 ± 0.12 mm vs. 0.41 ± 0.21 mm, *p* < 0.01), with a mean between-group difference of −0.18 mm (95% CI, −0.23 to −0.13 mm). Absolute Y-axis decentration was also significantly lower in the SMILE Pro group (0.13 ± 0.10 mm vs. 0.31 ± 0.22 mm, *p* < 0.01), with a mean between-group difference of −0.18 mm (95% CI, −0.23 to −0.13 mm). A box plot illustrating the distribution of absolute Y-axis decentration in the two groups is shown in Figure 1. Absolute X-axis decentration was lower in the SMILE Pro group (0.16 ± 0.12 mm vs. 0.20 ± 0.15 mm), although the difference did not reach statistical significance.

### Secondary outcomes: visual acuity and refractive outcomes

Postoperative clinical outcomes at 3 months, representing early-stage visual recovery and stability, are summarized in Table 3. There were no statistically significant differences between the SMILE group (VisuMax 500) and the SMILE Pro group (VisuMax 800) in postoperative IOP, UDVA, CDVA, efficacy index, or safety index (all *p* > 0.05).

**Table 3:** Comparison of postoperative visual acuity, refractive outcomes, and higher-order aberrations at 3 months between the SMILE and SMILE Pro groups.

Parameter	SMILE	SMILE Pro	*p*-value
IOP	13.30 ± 2.51	13.54 ± 1.93	0.450
Spherical	0.41 ± 0.46	0.22 ± 0.44	0.004
Cylindrical	−0.36 ± 0.24	−0.29 ± 0.24	0.048
SE	0.23 ± 0.46	0.07 ± 0.42	0.015
UDVA	1.16 ± 0.16	1.18 ± 0.11	0.208
CDVA	1.20 ± 0.12	1.19 ± 0.11	0.605
Efficacy index	0.97 ± 0.11	0.99 ± 0.10	0.302
Safety index	1.01 ± 0.12	0.99 ± 0.09	0.231
HOAs			
Spherical aberration	0.37 ± 0.02	0.36 ± 0.12	0.353
Horizontal coma	0.17 ± 0.11	0.16 ± 0.09	0.483
Vertical coma	0.25 ± 0.17	0.19 ± 0.12	0.004
Total HOAs	0.59 ± 0.12	0.56 ± 0.09	0.087

Data are presented as mean ± standard deviation (SD). IOP, intraocular pressure; D, diopters; SE, spherical equivalent; UDVA, uncorrected distance visual acuity (decimal); CDVA, corrected distance visual acuity (decimal); HOAs, higher-order aberrations. Statistical Analysis: Differences between groups were evaluated using independent two-sample *t*-tests. Statistically significant differences (*p* < 0.05) were observed in residual spherical error, cylindrical error, spherical equivalent, and vertical coma.

The postoperative cylindrical error was significantly lower in the SMILE Pro group than in the SMILE group (−0.29 ± 0.24 D vs. −0.36 ± 0.24 D, *p* = 0.048), although the absolute between-group difference was small (+0.07 D; 95% CI, 0.00 to 0.14 D). Postoperative spherical error and spherical equivalent were also closer to plano in the SMILE Pro group; however, the absolute intergroup differences were small.

**Figure 2:**
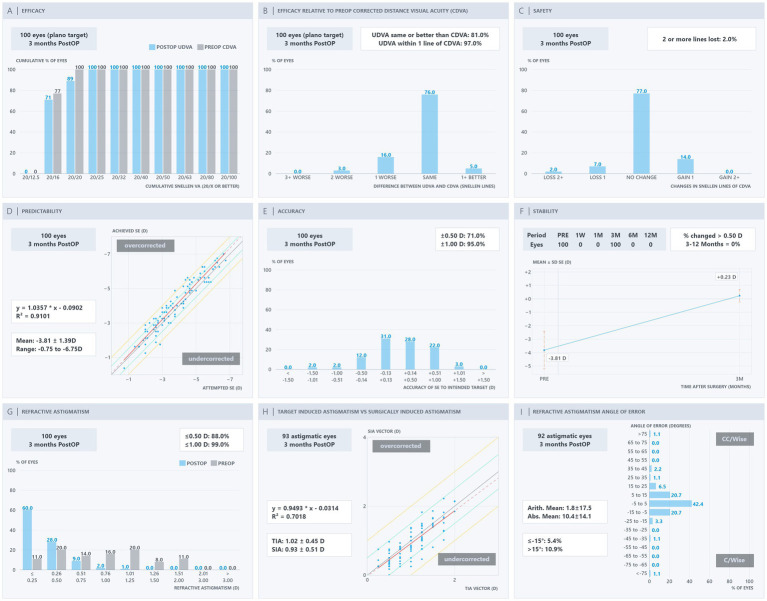
Standard refractive surgery outcomes for the SMILE group (VisuMax 500) at 3 months postoperatively.

**Figure 3:**
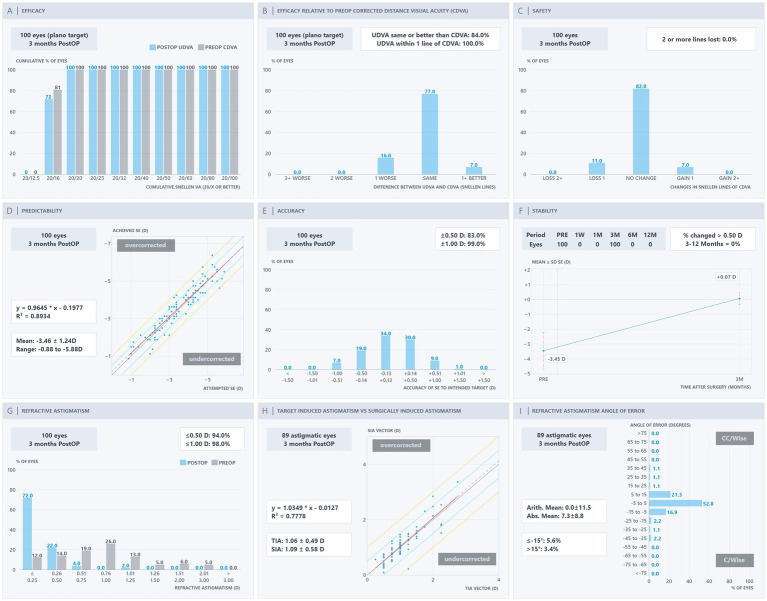
Standard refractive surgery outcomes for the SMILE Pro group (VisuMax 800) at 3 months postoperatively.

Standard nine-panel refractive outcome plots for the SMILE group (VisuMax 500) and SMILE Pro group (VisuMax 800) at 3 months are shown in Figures 2, 3, respectively. In terms of visual acuity outcomes, 100% of eyes in the SMILE Pro group achieved a UDVA of 20/20 or better, compared with 89% in the SMILE group. The proportion of eyes achieving a postoperative UDVA equal to or better than preoperative CDVA was 84% in the SMILE Pro group and 81% in the SMILE group. With respect to refractive accuracy, 83% of eyes in the SMILE Pro group were within ±0.50 D of the intended spherical equivalent target, compared with 71% in the SMILE group. Residual astigmatism of ≤0.50 D was achieved in 94% of eyes in the SMILE Pro group and 88% in the SMILE group. Due to the absence of earlier postoperative follow-up data, refractive stability plots reflect outcomes at the 3-month time point only.

### Secondary outcomes: higher-order aberrations

Regarding wavefront aberrations, no statistically significant differences were observed between the two groups in postoperative spherical aberration, horizontal coma, or total higher-order aberrations (HOAs) (all *p* > 0.05). In contrast, postoperative vertical coma was significantly lower in the SMILE Pro group (VisuMax 800) than in the SMILE group (0.19 ± 0.12 μm vs. 0.25 ± 0.17 μm, *p* = 0.004), with a mean between-group difference of −0.06 μm (95% CI, −0.10 to −0.02 μm), although the absolute difference was modest.

**Figure 4:**
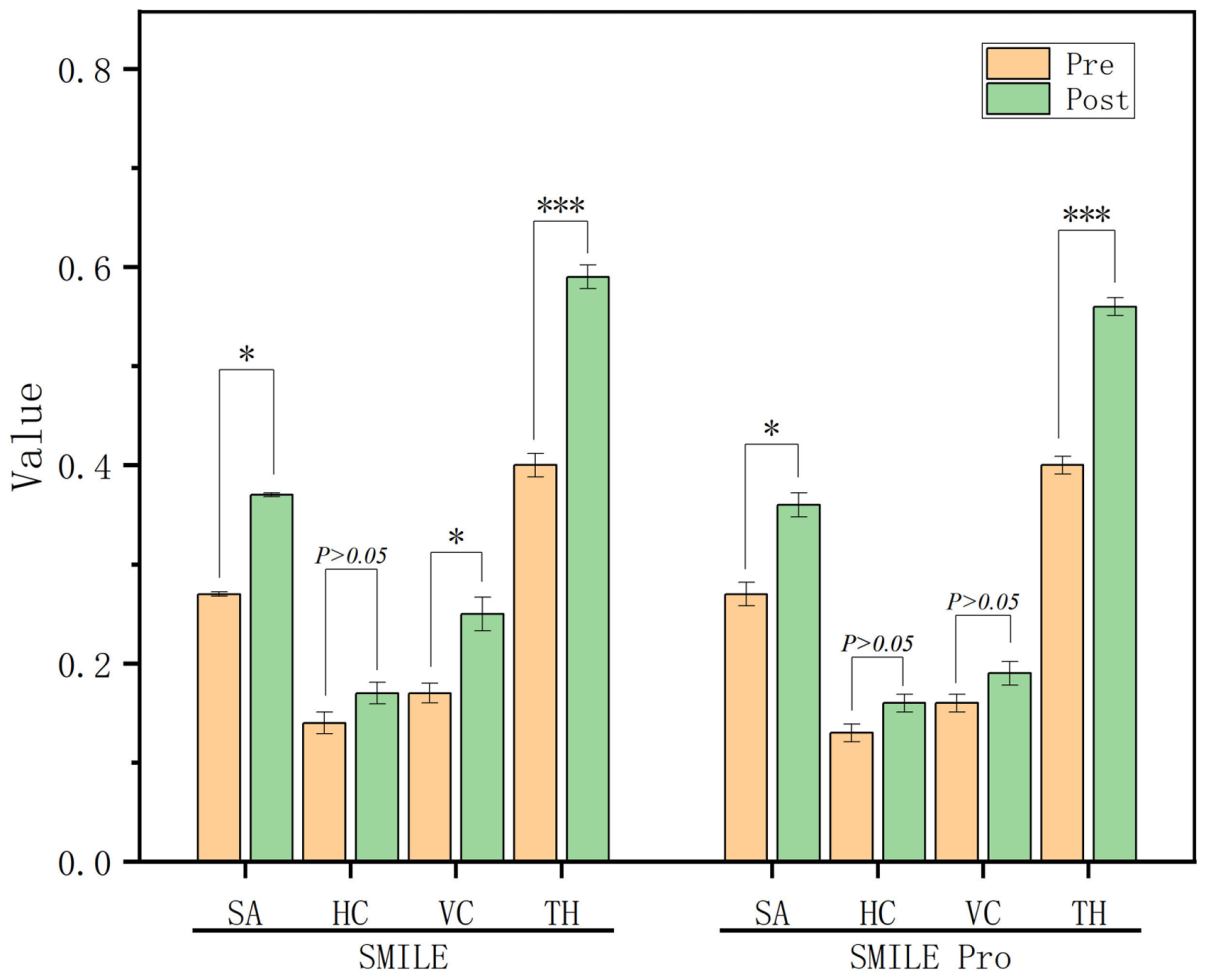
Comparison of preoperative and postoperative corneal higher-order aberrations (HOAs) between the SMILE and SMILE Pro groups. The bar chart illustrates the root mean square (RMS) values of spherical aberration, horizontal coma, vertical coma, and total HOAs.

Changes in higher-order aberrations from preoperative to postoperative measurements are illustrated in Figure 4. Both groups demonstrated a statistically significant increase in spherical aberration and total HOAs at 3 months compared with preoperative values (all *p* < 0.05). No statistically significant intergroup differences were observed in postoperative spherical aberration or horizontal coma. In contrast, a statistically significant increase in vertical coma was observed postoperatively in the SMILE group (*p* < 0.05), whereas no significant change was detected in the SMILE Pro group.

### Secondary outcomes: astigmatism vector analysis

Astigmatism vector analysis using the Alpins method was performed in eyes with preoperative astigmatism (SMILE: 93 eyes; SMILE Pro: 89 eyes). The results are summarized in Table 4. There were no significant between-group differences in target induced astigmatism (TIA), surgically induced astigmatism (SIA), difference vector (DV), correction index (CI), or angle of error (AE), although the mean AE tended to be smaller in the SMILE Pro group than in the SMILE group (7.30° ± 8.80° vs. 10.40° ± 14.10°, *p* = 0.077).

**Table 4:** Comparison of astigmatism vector analysis outcomes between the SMILE and SMILE Pro groups.

Vector parameter	SMILE (93)	SMILE Pro (89)	*p*-value
TIA	1.02 ± 0.45	1.06 ± 0.49	0.567
SIA	0.93 ± 0.51	1.09 ± 0.58	0.058
DV	0.37 ± 0.25	0.31 ± 0.24	0.101
ME	−0.08 ± 0.28	0.02 ± 0.27	0.015
AE	10.40 ± 14.10	7.30 ± 8.80	0.077
CI	0.93 ± 0.38	1.02 ± 0.28	0.072
IoS	0.44 ± 0.42	0.33 ± 0.26	0.036

Data are presented as mean ± standard deviation (SD). Astigmatism vector analysis was calculated using the Alpins method. TIA, target induced astigmatism; SIA, surgically induced astigmatism; DV, difference vector; ME, magnitude of error; AE, angle of error; CI, correction index; IoS, index of success. Statistical Analysis: Differences between groups were evaluated using independent two-sample *t*-tests. Statistically significant differences (*p* < 0.05) were observed in the Magnitude of Error (ME) and Index of Success (IoS).

The magnitude of error (ME) and index of success (IoS) were significantly lower in the SMILE Pro group compared with the SMILE group (ME: 0.02 ± 0.27 D vs. −0.08 ± 0.28 D, *p* = 0.015; IoS: 0.33 ± 0.26 vs. 0.44 ± 0.42, *p* = 0.036). The mean between-group difference was +0.10 D (95% CI, 0.02 to 0.18 D) for ME and −0.11 (95% CI, −0.21 to −0.01) for IoS.

**Figure 5:**
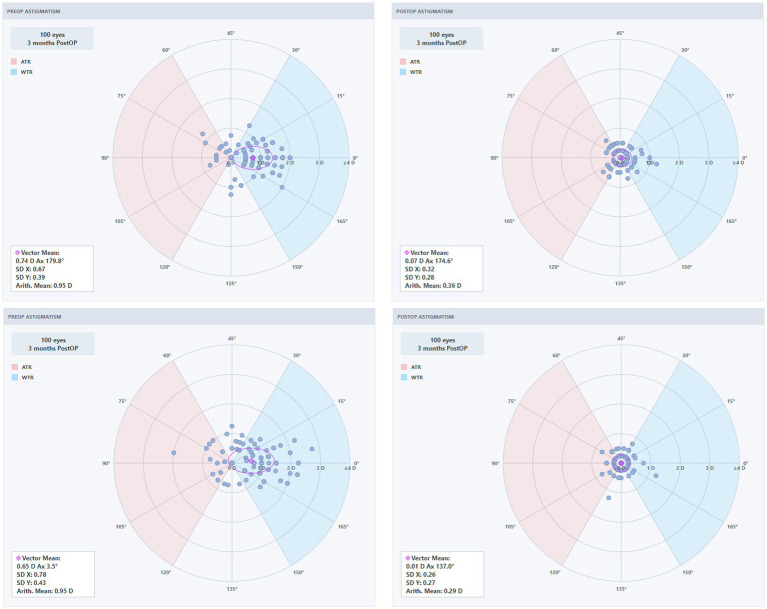
Astigmatism vector analysis polar plots for the SMILE group (VisuMax 500).

**Figure 6:**
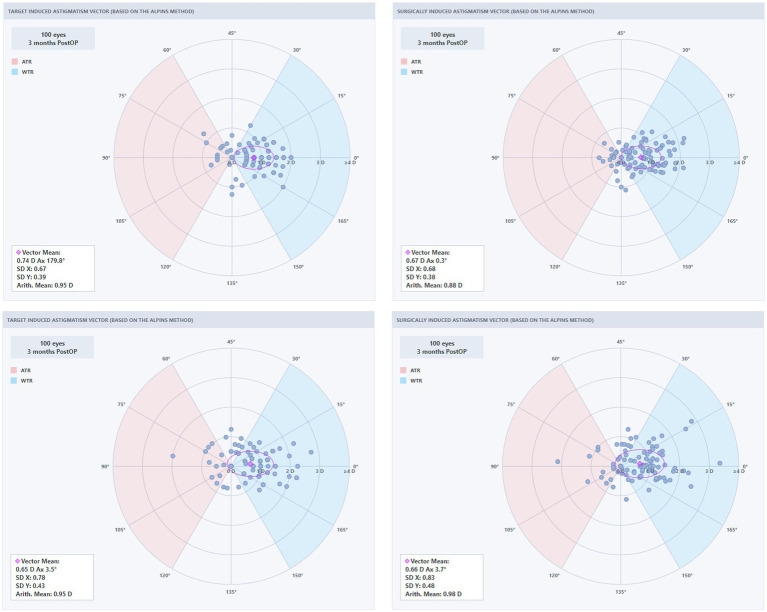
Astigmatism vector analysis polar plots for the SMILE Pro group (VisuMax 800).

To further illustrate the astigmatic outcomes, polar plots were generated to visualize the distributions of astigmatism vectors before and after surgery, as well as the relationship between target induced astigmatism and surgically induced astigmatism (Figures 5, 6). Preoperatively, the two groups showed broadly similar distributions of astigmatism vectors. Postoperatively, residual astigmatism vectors in both groups were more concentrated around the origin, indicating reduced residual astigmatism after surgery. This distribution appeared visually more compact in the SMILE Pro group; however, these plots are descriptive and should be interpreted together with the quantitative vector parameters shown in Table 4.

### Exploratory analyses

**Figure 7:**
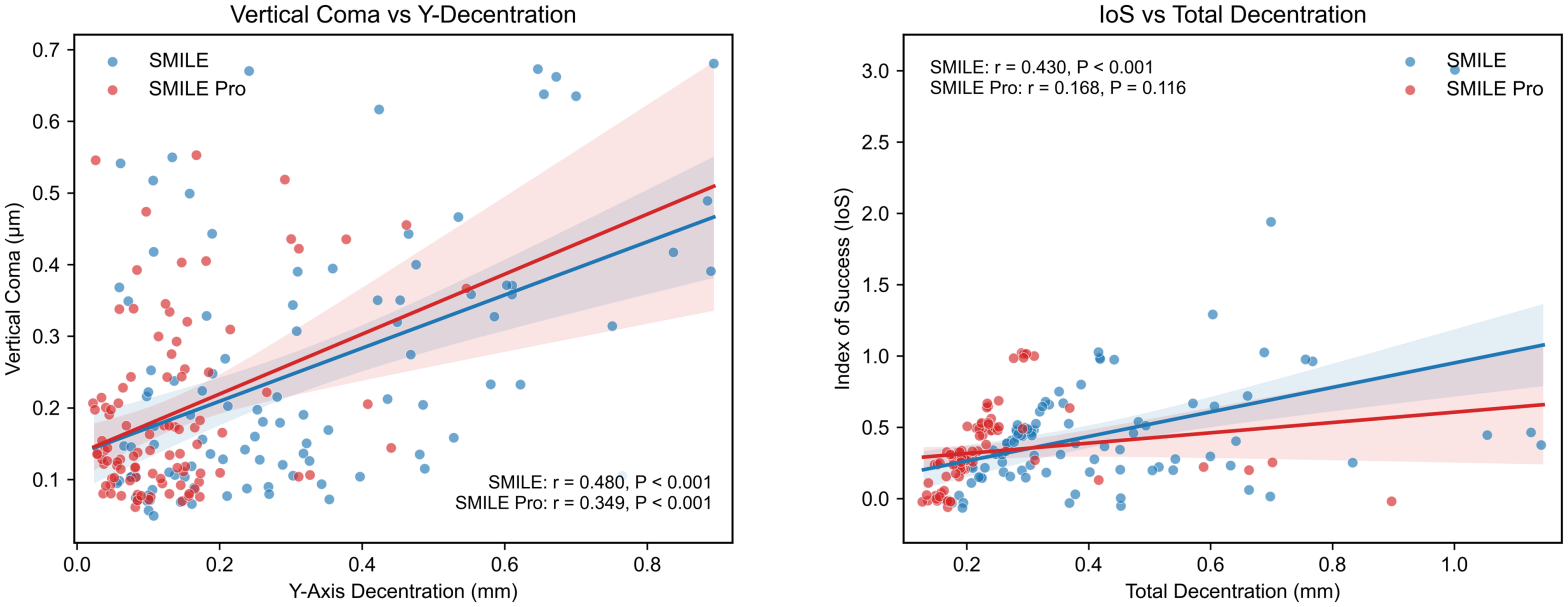
Pearson correlation analysis between centration accuracy and postoperative visual metrics. The scatter plots depict the relationship between absolute Y-axis decentration and vertical coma (Left), and between total decentration and the Index of Success (Right).

Exploratory Pearson correlation analyses are presented in Table 5 and Figure 7. Absolute Y-axis decentration was significantly positively correlated with postoperative vertical coma in both groups (SMILE: *r* = 0.48, *p* < 0.001; SMILE Pro: *r* = 0.349, *p* = 0.0015). In addition, total decentration was significantly positively correlated with the index of success in the SMILE group (*r* = 0.43, *p* < 0.001), but not in the SMILE Pro group (*r* = 0.168, *p* = 0.116). These findings indicate a between-group difference in the observed association between decentration and astigmatic vector outcomes; however, this observation should be interpreted cautiously because cyclotorsion was not directly measured in the present study.

**Table 5:** Pearson correlation analysis between intraoperative decentration parameters and postoperative visual outcomes in the SMILE and SMILE Pro groups.

Group	Variable pair	*r*	*t*	*p*-value
SMILE	Vertical coma vs. Y-decentration	0.48	4.83	<0.001
SMILE Pro	Vertical coma vs. Y-decentration	0.349	3.29	0.0015
SMILE	IOS vs. total decentration	0.43	4.21	<0.001
SMILE Pro	IOS vs. total decentration	0.116	1.51	0.136

IoS, index of success; *r*, Pearson correlation coefficient. Statistical Analysis: Pearson correlation analysis was performed to evaluate the associations between centration metrics and visual outcomes.

## Discussion

This retrospective comparative study evaluated early clinical outcomes and optical quality–related parameters after SMILE performed with VisuMax 500 and VisuMax 800 in a Chinese population with myopia and low-to-moderate astigmatism. The principal finding of this study was that the two platforms showed comparable safety and overall visual acuity outcomes, whereas SMILE Pro was associated with more favorable astigmatic vector outcomes and lower postoperative vertical coma.

Though statistically significant difference between the two groups of comparison in terms of spherical equivalent and spherical correction was established, the degree of the differences was very low and they could not be translated into any meaningful difference in postoperative uncorrected distance visual acuity (11). This could be partly attributed to the fact that the population used in the study is still young and that in this case the accommodative reserve may be able to counter the low residual hyperopic error (12, 13).

Among the notable technical breakthroughs of SMILE Pro (VisuMax 800) platform, the significant shortening of the lenticule creation time is noteworthy due to the increased laser repetition rate. In the present study, the lenticule creation time in the SMILE Pro group was reduced to less than 10 s.

A shorter laser scanning time may have clinical implications beyond procedural efficiency. It has been found that prolonged suction time also results in more patient discomfort, and more chances of having involuntary eye movements which may impair the judgment of centration (14). Also, past research has proposed that the hydration condition of the corneal stroma can affect laser-tissue interaction and refractive performance (15–17). Although the stromal hydration was not directly measured in the current study, the decreased exposure period with SMILE Pro could be partially involved in the trends of refractive indices. Stromal hydration quantitative studies are required in the future to confirm this hypothesis.

Both SMILE (VisuMax 500) and SMILE Pro (VisuMax 800) resulted in an increase in total higher-order aberrations and spherical aberration, consistent with previous reports on corneal refractive surgery (18, 19). These changes are likely attributable to alterations in corneal asphericity and postoperative wound healing rather than platform-specific effects.

Although some between-group differences reached statistical significance, their absolute magnitudes were small. In particular, the differences in postoperative residual cylinder and vertical coma were modest, and whether these differences translate into meaningful patient-perceived visual benefit remains uncertain.

In contrast, postoperative vertical coma differed between the two platforms. Vertical coma increased significantly after conventional SMILE but remained relatively stable following SMILE Pro. Given that vertical coma is sensitive to vertical decentration of the optical zone, this finding may reflect a difference in centration-related optical outcomes between the two platforms. However, the absolute between-group difference was small, and its direct clinical relevance should be interpreted with caution.

Astigmatism vector analysis showed lower magnitude of error and index of success values in the SMILE Pro group (VisuMax 800) than in the conventional SMILE group (VisuMax 500), suggesting better overall astigmatic vector outcomes in this cohort. However, these findings should not be interpreted as indicating that improved translational centration alone led to better astigmatic correction. From an optical and surgical perspective, astigmatic correction accuracy may be influenced by both translational alignment and rotational factors, particularly cyclotorsion.

In the present study, CentraLign was used for centration guidance and OcuLign cyclotorsion compensation was enabled in all SMILE Pro eyes. However, cyclotorsion was not directly quantified. Therefore, the between-group difference in astigmatic vector outcomes cannot be attributed solely to centration accuracy, and the underlying mechanism should be interpreted with caution.

By contrast, the correlation analysis demonstrated that vertical decentration was significantly associated with postoperative vertical coma in both groups, which is more directly consistent with the known optical consequences of decentration. In addition, only the SMILE group showed a significant correlation between total decentration and the index of success, whereas this association was not observed in the SMILE Pro group. This may suggest a difference in how decentration-related factors were associated with astigmatic vector outcomes between the two platforms within the observed range; however, this observation remains exploratory and should not be overinterpreted.

Clinically, the present findings suggest that SMILE Pro may provide favorable centration-related, aberration-related, and astigmatic vector outcomes in eyes with low-to-moderate astigmatism. The shorter laser scanning time may also be advantageous in patients with less stable fixation or greater intraoperative anxiety.

In addition, because subtle differences in refractive and astigmatic vector outcomes were observed between platforms, further evaluation of platform-specific nomogram optimization may be warranted.

This study has several limitations. First, it was a retrospective study rather than a randomized controlled trial (20, 21). Although both platforms were used concurrently during the same study period and all procedures were performed by a single experienced surgeon, potential selection bias and residual learning-curve effects could not be fully excluded. In addition, this was a single-center study conducted in a Chinese population, which may limit the generalizability of the findings to other clinical settings and ethnic populations.

Second, the follow-up period was limited to 3 months. Although some previous studies have suggested that refractive outcomes after SMILE may stabilize relatively early (7), standard refractive surgery reporting commonly includes 6- to 12-month follow-up to better assess long-term refractive stability (22). Therefore, the present results should primarily be interpreted as early postoperative outcomes rather than definitive evidence of long-term stability. Further studies with longer follow-up are needed to confirm the long-term refractive and optical performance of the VisuMax 800 platform.

Third, we purposefully restricted preoperative cylindrical error to ≤2.00 D. While this limits the generalizability of our findings to high astigmatism cases, this criterion was established to minimize the confounding impact of static cyclotorsion errors, which are more critical in high astigmatism corrections (23). This criterion also helped reduce the potential confounding effect of larger astigmatic corrections when interpreting centration-related outcomes (24). The potential value of rotational compensation features in eyes with higher astigmatism warrants further investigation in future studies.

Fourth, the exploratory correlation analyses were limited to the relationships between vertical coma and Y-axis decentration, and between the index of success (IoS) and total decentration. It should be mentioned that IoS is a composite measure and depends on the axis correction, as well as the magnitude (23). The present study did not separately evaluate the influence of total decentration on individual vector components, such as magnitude of error (ME) or angle of error (AE), which limited a more detailed understanding of how decentration may affect specific dimensions of astigmatic correction. In addition, the statistical model was based primarily on univariate correlation analyses rather than multivariate regression models. This limited control of potential confounding factors and prevented any conclusive inference regarding causal relationships between intraoperative parameters and postoperative visual outcomes (25). Multivariate regression analysis correcting possible confounding factors like pupil diameter, optical zone size and preoperative astigmatism were not done limiting the causal inference (26). Additionally, repeatability of decentration measurements was not formally assessed in this retrospective study, which should be considered when interpreting the centration-related findings; moreover, this observation should be interpreted cautiously because cyclotorsion was not directly measured in the present study.

Fifth, visual quality is multifactorial and cannot be fully captured by corneal higher-order aberrations alone. In the present study, postoperative HOAs were evaluated as objective optical quality–related parameters; however, other important aspects of visual quality, including contrast sensitivity, optical scatter, glare/halo symptoms, and patient-reported quality-of-vision outcomes, were not assessed. Therefore, the findings related to aberrations should be interpreted cautiously and should not be considered a comprehensive evaluation of visual quality.

## Conclusion

Both VisuMax 500- and VisuMax 800-based SMILE provided favorable early safety and efficacy for the correction of myopia and low-to-moderate astigmatism. Compared with conventional SMILE, SMILE Pro was associated with shorter laser scanning time, lower optical zone decentration, lower postoperative vertical coma, and more favorable astigmatic vector outcomes. These findings represent early postoperative results and should be interpreted cautiously.

## Data Availability

The original contributions presented in the study are included in the article/Supplementary material, further inquiries can be directed to the corresponding authors.

## References

[1] Wang Y Xie L Yao K Sekundo W Alió JL Mehta JS . Evidence-based guidelines for Keratorefractive Lenticule extraction surgery. Ophthalmology. (2025) 132:397–419. doi: 10.1016/j.ophtha.2024.11.016, 39577672

[2] Sekundo W Kunert K Russmann C Gille A Bissmann W Stobrawa G . First efficacy and safety study of femtosecond lenticule extraction for the correction of myopia: six-month results. J Cataract Refract Surg. (2008) 34:1513–20. doi: 10.1016/j.jcrs.2008.05.033, 18721712

[3] Shah R Shah S Sengupta S. Results of small incision lenticule extraction: all-in-one femtosecond laser refractive surgery. J Cataract Refract Surg. (2011) 37:127–37. doi: 10.1016/j.jcrs.2010.07.033, 21183108

[4] Reinstein DZ Archer TJ Gobbe M. Small incision lenticule extraction (SMILE) history, fundamentals of a new refractive surgery technique and clinical outcomes. Eye Vis (Lond). (2014) 1:3. doi: 10.1186/s40662-014-0003-1, 26605350 PMC4604118

[5] Reinstein DZ Archer TJ Randleman JB. Mathematical model to compare the relative tensile strength of the cornea after PRK, LASIK, and small incision lenticule extraction. J Refract Surg. (2013) 29:454–60. doi: 10.3928/1081597X-20130617-03, 23820227

[6] Ivarsen A Asp S Hjortdal J. Safety and complications of more than 1500 small-incision lenticule extraction procedures. Ophthalmology. (2014) 121:822–8. doi: 10.1016/j.ophtha.2013.11.006, 24365175

[7] Blum M Täubig K Gruhn C Sekundo W Kunert KS. Five-year results of small incision Lenticule extraction (ReLEx SMILE). Br J Ophthalmol. (2016) 100:1192–5. doi: 10.1136/bjophthalmol-2015-306822, 26746577

[8] Zhao J He L Yao P Shen Y Zhou Z Miao H . Diffuse lamellar keratitis after small-incision lenticule extraction. J Cataract Refract Surg. (2015) 41:400–7. doi: 10.1016/j.jcrs.2014.05.041, 25661134

[9] Reinstein DZ Archer TJ Potter JG Gupta R Wiltfang R. Refractive and visual outcomes of SMILE for compound myopic astigmatism with the VISUMAX 800. J Refract Surg. (2023) 39:294–301. doi: 10.3928/1081597X-20230301-02, 37162399

[10] Kim BK Chung YT. Comparison of clinical outcomes following small incision lenticule extraction performed with the visumax 800 versus visumax 500 femtosecond laser. Sci Rep. (2025) 15:25484. doi: 10.1038/s41598-025-98041-9, 40664759 PMC12264011

[11] Wen D McAlinden C Flitcroft I Tu R Wang Q Alió J . Postoperative efficacy, predictability, safety, and visual quality of laser corneal refractive surgery: a network meta-analysis. Am J Ophthalmol. (2017) 178:65–78. doi: 10.1016/j.ajo.2017.03.013, 28336402

[12] Jorge J Queiros A González-Méijome J Fernandes P Almeida JB Parafita MA. The influence of cycloplegia in objective refraction. Ophthalmic Physiol Opt. (2005) 25:340–5. doi: 10.1111/j.1475-1313.2005.00277.x, 15953119

[13] Meng C Zhang Y Wang S. Changes in accommodation and convergence function after refractive surgery in myopic patients. Eur J Ophthalmol. (2023) 33:29–34. doi: 10.1177/11206721221128993, 36203367

[14] Osman IM Awad R Shi W Shousha MA. Suction loss during femtosecond laser-assisted small-incision lenticule extraction: incidence and analysis of risk factors. J Cataract Refract Surg. (2016) 42:246–50. doi: 10.1016/j.jcrs.2015.10.067, 27026449

[15] Patel S Alió JL Pérez-Santonja JJ. Refractive index change in bovine and human corneal stroma before and after lasik: a study of untreated and re-treated corneas implicating stromal hydration. Invest Ophthalmol Vis Sci. (2004) 45:3523–30. doi: 10.1167/iovs.04-0179 15452058

[16] Feltham MH Stapleton F. The effect of water content on the 193 nm excimer laser ablation. Clin Experiment Ophthalmol. (2002) 30:99–103. doi: 10.1046/j.1442-6404.2002.00496.x, 11886412

[17] Kim WS Jo JM. Corneal hydration affects ablation during laser in situ keratomileusis surgery. Cornea. (2001) 20:394–7. doi: 10.1097/00003226-200105000-00011, 11333327

[18] Miao H Han T Tian M Wang X Zhou X. Visual quality after femtosecond laser small incision Lenticule extraction. Asia Pac J Ophthalmol (Phila). (2017) 6:465–8. doi: 10.22608/APO.2016171, 28379651

[19] Sekundo W Gertnere J Bertelmann T Solomatin I. One-year refractive results, contrast sensitivity, high-order aberrations and complications after myopic small-incision lenticule extraction (ReLEx SMILE). Graefes Arch Clin Exp Ophthalmol. (2014) 252:837–43. doi: 10.1007/s00417-014-2608-4, 24647595

[20] Grimes DA Schulz KF. Bias and causal associations in observational research. Lancet. (2002) 359:248–52. doi: 10.1016/S0140-6736(02)07451-2 11812579

[21] Burns PB Rohrich RJ Chung KC. The levels of evidence and their role in evidence-based medicine. Plast Reconstr Surg. (2011) 128:305–10. doi: 10.1097/PRS.0b013e318219c171, 21701348 PMC3124652

[22] Waring GO 3rd. Standard graphs for reporting refractive surgery. J Refract Surg. (2000) 16:459–66. 10939727

[23] Alpins N. Astigmatism analysis by the Alpins method. J Cataract Refract Surg. (2001) 27:31–49. doi: 10.1016/S0886-3350(00)00798-7 11165856

[24] Lee H Roberts CJ Arba-Mosquera S Kang DSY Reinstein DZ Kim TI. Relationship between Decentration and induced corneal higher-order aberrations following small-incision Lenticule extraction procedure. Invest Ophthalmol Vis Sci. (2018) 59:2316–24. doi: 10.1167/iovs.17-23451, 29847636

[25] Katz MH. Multivariable analysis: a primer for readers of medical research. Ann Intern Med. (2003) 138:644–50. doi: 10.7326/0003-4819-138-8-200304150-00012, 12693887

[26] Oshika T Tokunaga T Samejima T Miyata K Kawana K Kaji Y. Influence of pupil diameter on the relation between ocular higher-order aberration and contrast sensitivity after laser in situ Keratomileusis. Invest Ophthalmol Vis Sci. (2006) 47:1334–8. doi: 10.1167/iovs.05-1154, 16565365

